# A Functional Model for Unifying Brain Computer Interface Terminology

**DOI:** 10.1109/OJEMB.2021.3057471

**Published:** 2021-02-05

**Authors:** Chuck Easttom, Luigi Bianchi, Davide Valeriani, Chang S. Nam, Ali Hossaini, Dariusz Zapała, Avid Roman-Gonzalez, Avinash K Singh, Alberto Antonietti, Guillermo Sahonero-Alvarez, Pradeep Balachandran

**Affiliations:** Georgetown University 8368 Washington DC 20057 USA; Tor Vergata University Rome 00133 Italy; Harvard University 1857 Boston MA 02114 USA; North Carolina State University 6798 Raleigh NC 27695 USA; King's College London 4616 London N6 6HD U.K.; John Paul II Catholic University of Lublin 49642 Lublin 20-950 Poland; Universidad Nacional Tecnologica de Lima Sur 15834 Villa el Salvador Peru; Australian Artificial Intelligence InstituteUniversity of Technology 1994 Sydney NSW 2007 Australia; Politecnico di Milano 18981 20133 Milan Italy; Universidad Católica Boliviana San Pablo 28140 4805 La Paz Bolivia

**Keywords:** BCI functional models, BCI Lexicography, brain computer interface, brain mind interface

## Abstract

Brain Computer Interface (BCI) technology is a critical area both for researchers and clinical practitioners. The IEEE P2731 working group is developing a comprehensive BCI lexicography and a functional model of BCI. The glossary and the functional model are inextricably intertwined. The functional model guides the development of the glossary. Terminology is developed from the basis of a BCI functional model. This paper provides the current status of the P2731 working group's progress towards developing a BCI terminology standard and functional model for the IEEE.

## I. Introduction

During the last few decades, there has been an emergence of new capabilities by which the human brain can directly communicate with the environment. The technology is referred to by several synonymous terms: brain–computer interface (BCI), brain–machine interface (BMI), direct neural interface, or mind–machine interface (MMI). For the purposes of this paper, the authors will use the term BCI. BCIs enable direct communication with a system via brain signals for both medical applications [1] and research applications.

What is currently missing from the research literature is a comprehensive BCI research paradigm. A comprehensive research paradigm enables the evaluation and verification of results published in the scientific literature [2]. Factors such as incomplete information on the procedure, the data processing methods, and analysis processes in published reports make successful replication or comparison of research problematic [3]. There are meta-analyses studies that have noted that a significant number of BCI publications lack necessary information [4], [5]. This lack of detail can have multiple deleterious effects. One such effect, is that it can lead to a perceived heterogeneity of results [5] which may not be accurate. Furthermore, the lack of detail can make it challenging to compare results obtained in different studies [6]. For example, in a meta-analysis and literature review of studies on signal processing in motor imagery BCI, 55 of 131 articles did not provide sufficient statistics to support effective meta-analysis [5]. A comprehensive research paradigm can ameliorate some of these issues.

Wierzgała and colleagues [3] suggest that the main reason for the low quality of many BCI publications is the lack of unified standards for a research paradigm description. Marchetti and Priftis [5] also recommended more detailed reporting in BCI papers, particularly by providing information about participants' cognitive and sensory state. Despite these suggestions, there is no unified paradigm current methodological literature that should be included in BCI publications.

One method to improve this situation is a commonly accepted glossary. By ensuring standardized communications, it is at least clear what diverse researchers mean when using particular terminology. Another method to improve the disparity in research results is a functional model of BCI. Having a clear model of brain computer interfaces, provides a common paradigm from which to conduct research and to communicate results. A third technique for improving research publications is the establishment of data sharing standards. These are the goals of the IEEE P2731 working group.

In addition to the current paper, the supplementary materials contains additional information. In the supplementary materials can be found additional diagrams of the control model, more details on specific BCI implementations, additional details on transducers, and data on benchmarking BCI's.

THe IEEE P2731 working group is currently working on standards for BCI terminology. For the terminology to have a practical benefit for researchers, such terminology must be related to a specific framework [6], [7]. The P2731 working group has been taking the approach of simultaneously formulating a functional model along with a BCI terminology lexicon. The working group's activity can be divided into three main areas:

1) the creation of a BCI glossary, which includes hundreds of terms, each of them with references and definitions. Moreover, multiple definitions for the same term could be provided to be comprehended according to each BCI stakeholder background (e.g., a user vs an engineer vs a neurologist);
2) The definition of a standard BCI functional model, which can take care of all the relevant aspects of a BCI;
3) The identification of the information that should be stored into data files to allow an easy and painless sharing of resources (data and tools).

There are existing publications that define subsets of terminology related to BCI. Neuroanatomy and physiology dictionaries are well known [8]–[10]. These lexicons are consulted in the development of the IEEE P2731 standard and many of their terms will become integrated in that standard. However, what is missing from the current literature is a comprehensive lexicon for BCI. Such a nomenclator would facilitate research efforts as well as the work of clinical practitioners. By having common terminology researchers and clinicians can more readily share data.

It should be noted that referring to the P2731 activity throughout the manuscript, it is a work-in-progress and the final standard could differ from what is described here. These changes would reflect inputs and suggestions from several sources, including readers of this paper.

## II. Review of Literature

There certainly have been other attempts to create a BCI glossary. As early as 2003, Mason and Birch published a general framework for BCI design [11]. There has also been effort to establish commonality in benchmarking [12], reporting [13], and protocols for bio signal transmissions [14]. As early as 2003, Mason and Birch [15] suggested a framework for BCI design. This work was an important step in the right direction, but the authors acknowledged it was only a partial step and more work was needed.

Each of these studies did contribute significantly to providing a common framework for BCI research. However, each was focused on a rather narrow application. None of these previous studies provided a commonly applicable model. Furthermore, a model that is part of an IEEE standard would be more likely to be widely adopted, thus the need for the model being developed by the IEEE 2731 working group.

## III. Functional Model

The importance of having a comprehensive functional model that can apply to virtually any BCI is that the same terminology, the same description, and the same language can be used even if different paradigms and applications are being discussed. It will be then easier to propose standard procedures such as benchmarking systems.

**Fig. 1.**
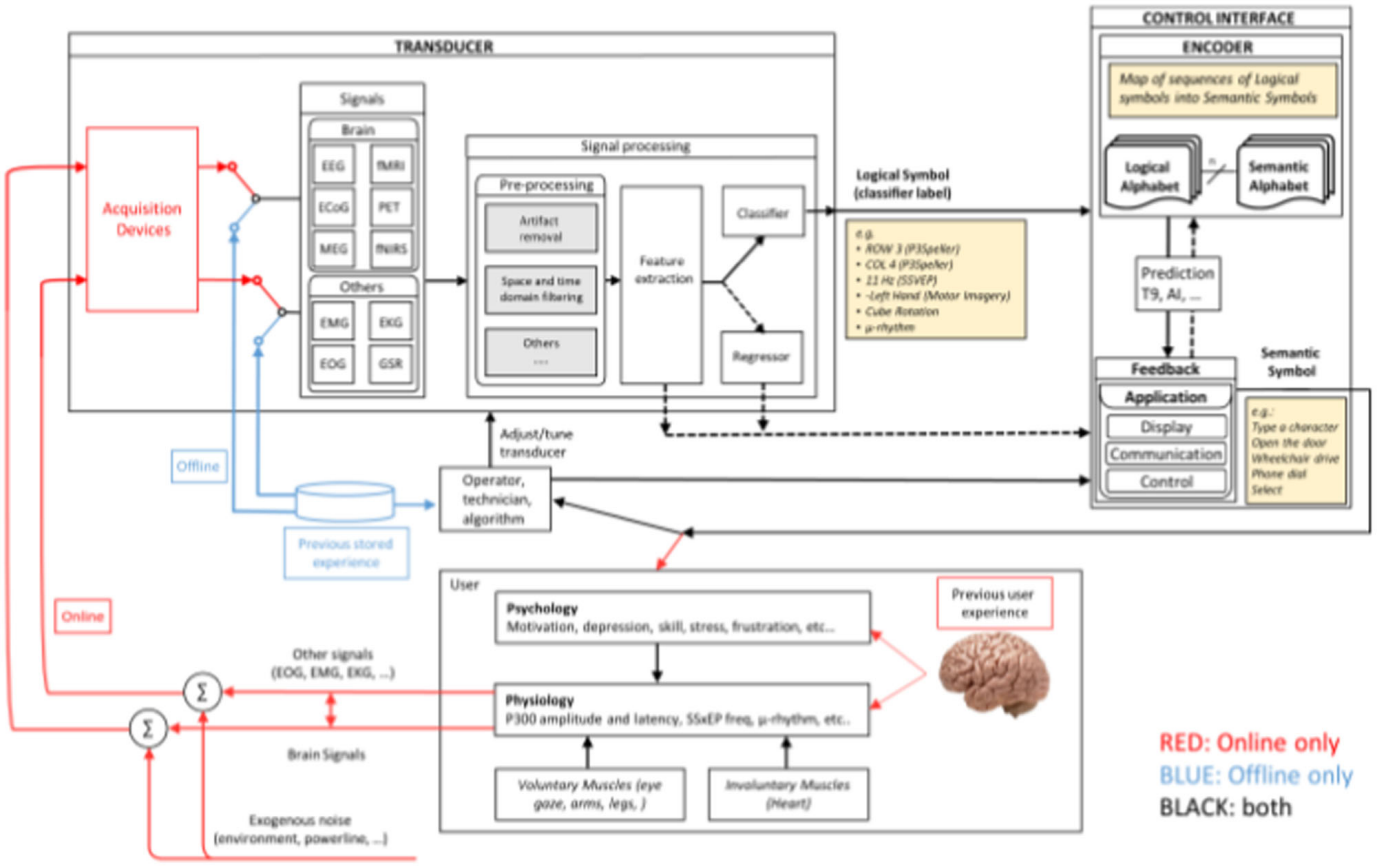
Functional Model.

Fig. 1 provides a general overview of the functional model being used by the IEEE 2731 working group. Additional details are available in the supplemental materials to this paper. Note, the figure can be seen in larger size in the supplemental material, and subsections are highlighted later in this paper.

The model addresses the BCI process first by defining large categories such as the transducer, control interface, physiology, and psychology. These are then further sub-divided. By associating the lexicography with this functional model, the resultant glossary is more effective for researchers. In the following sub sections, the main categories are described.

### A. Transducer

**Fig. 2.**
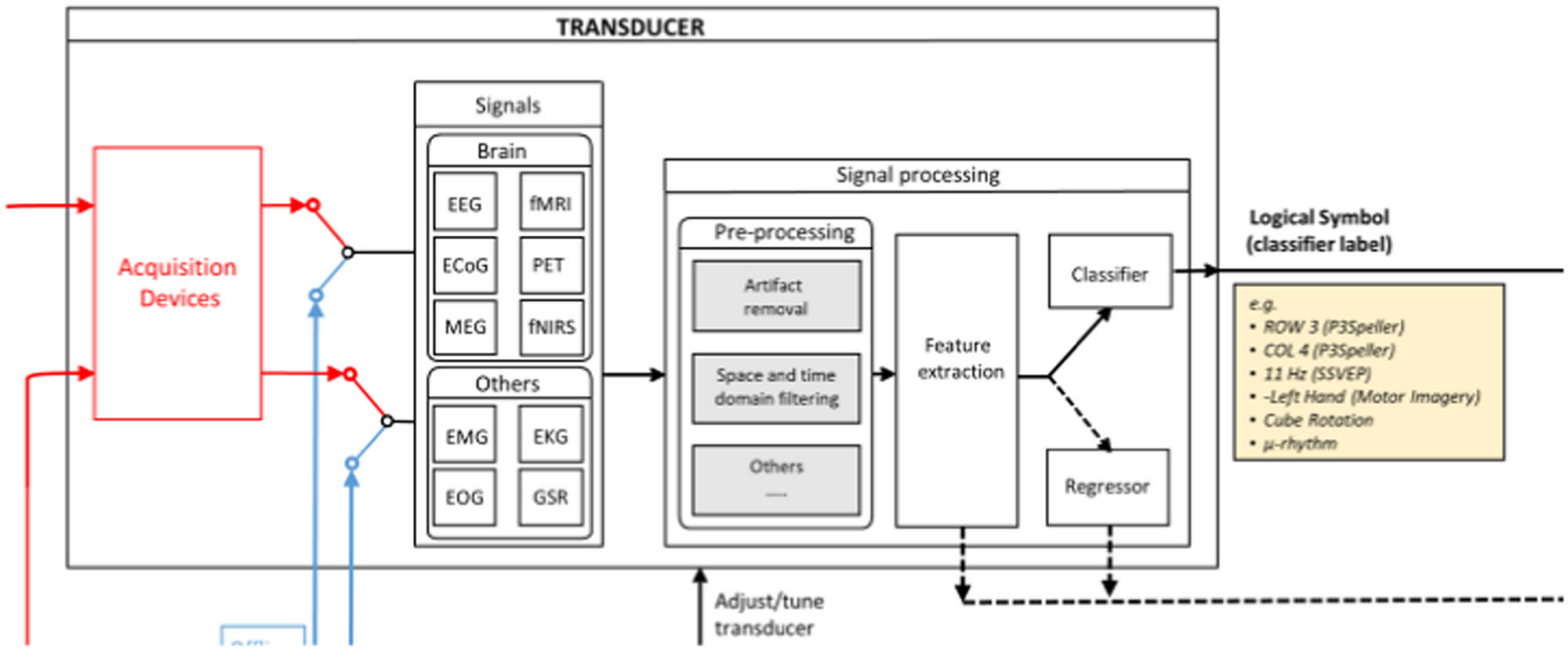
Transducer.

**Fig. 3.**
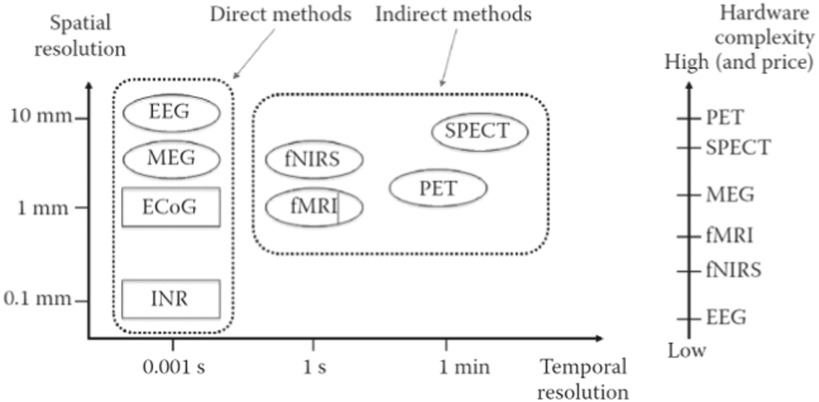
A comparison of noninvasive (oval) and invasive (rectangular) recording methods for use in BCIs based on temporal resolution (x-axis) and spatial resolution (y-axis); The x-axis and y-axis scales are not evenly distributed. (adopted from Nam *et al.* (2018) with permission from CRC Press).

Fig. 2 shows the transducer portion of the function model, which is mainly composed of three main processing stages: (1) a signal processing stage, where the recorded data is preprocessed; (2) a feature extraction stage, where meaningful neural information is extracted from the recorded data; and (3) a classification stage, where a user's intention is decoded from the neural data. Note that in figure two the acquisition devices are placed inside the transducer box. This is to signify that the signal acquisition sends data to the transducer, not that the acquisition device is actually part of the transducer. Signal acquisition is discussed separately in reference to Fig. 3.

Acquired signals include electroencephalograph, functional magnetic resonance imaging, positron emission tomography, electro-corticography, magnetoencephalography, and functional near-infrared spectroscopy. Understanding the diverse mechanisms for signal acquisition naturally leads to the description of a host of related terminology.

As was discussed earlier in this paper, this is a work in progress, and the working model will continue to evolve. For example, single-photon emission computed tomography [11] will be included in the signal acquisition model.

The output of the transducer is a Logical Symbol (LS) which corresponds to a classifier label and represents the identification of brain pattern. The set of LSs that can be outputted by a Transducer is called Logical Alphabet. An example of it can be a row or a column of a P3Speller or a detected frequency in an SSVEP (Steady State Visually Evoked Potential) as well as the identification of a mental task, such as the imagination of a hand movement. It is important to underline that the Transducer is the module that is responsible to process brain signals and detect paradigm-specific patterns.

#### 1) Signal Acquisition Stage

BCIs have used various neuroimaging methods acquire one's brain signals, often categorized by their invasiveness, spatial/temporal resolution, direct/indirect measurement, and complexity/price (see Fig. 3). Each recording technique has strengths, weaknesses, and specific uses that help BCI researchers decide which method is the best for their research goal.

Noninvasive recording methods are neuroimaging techniques in which sensors (e.g., wet or dry electrodes) are placed on the scalp. Two types of noninvasive recording methods have been widely used: (1) direct measures that detect electrical (e.g., electroencephalography, EEG) or magnetic activity (e.g., magnetoencephalography, MEG) of the brain, and (2) indirect measures of brain function reflecting brain metabolism or hemodynamics of the brain such as functional magnetic resonance imaging (fMRI), functional near infrared spectroscopy (fNIRS) and positron emission tomography (PET). In addition, direct brain monitoring approaches typically provide detailed information with high temporal resolution, but they normally lack spatial coverage; indirect measures show higher spatial resolution that direction measures.

Unlike noninvasive recording methods, invasive approaches require surgery, machine implantation, or needle insertion to directly acquire neural activity [16]. Two types of invasive recording methods have been widely adopted by the BCI community: electrocorticography (ECoG) and intracortical neuron recording (INR).

#### 2) Signal Processing Stage

The raw signals recorded from the brain in the signal acquisition stage often contain other information, known as ‘noisy signals’ or ‘artifact’ which are added by environmental (e.g., power line) and physiological sources. This extraneous data has been known to hinder BCI performance, and thus it is important to remove noise for the recorded signal.

To enhance sensitivity to particular brain sources and improve source localization, two main types of spatial filtering methods have been commonly used by BCI researchers: referencing and common special patterns. Referencing can be further subdivided based on specific techniques such as common average reference (CAR), surface (small or large) Laplacian, and bipolar reference and data-dependent spatial filters such as principal component analysis (PCA), independent component analysis (ICA), and common spatial patterns (CSP) [17].

#### 3) Feature Extraction Stage

Feature extraction is usually part of the signal processing block. However, given the importance of this process, we have separated its description into a separate section of the paper. Feature extraction is the process of distinguishing and identifying the pertinent signal characteristics from raw neural data [18], [19]. Examples include event-related potential (ERP) components such as N2pc, N400, P300 and the error related negativity (ERN), event-related (de) synchronization (ERD/S) steady state visually evoked potentials (SSVEP), Somatosensory Evoked Potential (SSEP), Local Field Potential (LFP) [20] and sensorimotor rhythm (SMR).

The main goal of feature extraction is to make it easier to identify patterns and improve the accuracy of the BCI using supervised or unsupervised methods. Another related goal is data dimensionality reduction. Major types of feature extraction methods include time and/or frequency methods, spatial filtering methods and dimensional reduction methods. Some feature extraction methods such as Principal Component Analysis (PCA) and Wavelet transform are unsupervised in that they do not use example data labelled with features. Other methods like Common Spatial Patterns (CSP) and adaptive autoregressive parameters (AARP) are supervised, which require a set of labelled data to extract the specific patterns of interests.

#### 4) Classification Stage

The next stage of the functional model is to decode a BCI user's intention by classifying brain features extracted in the feature extraction stage. Many different classification techniques have been used for BCIs, such as linear classifiers (e.g., linear discriminant analysis, LDA; support vector machine, SVM), artificial neural network (ANN) classifiers, and hidden Markov model (HMM) classifiers.

The signal processing module includes components to transform the neural data into a BCI output. First, designers have to decide whether the BCI will be operating online/real-time or offline. Next, the pre-processing submodule includes the application of signal processing techniques to increase the signal-to-noise ratio (SNR) of the neural data by rejecting artifacts and performing spatio-temporal filtering. Techniques used in this stage include electrooculogram (EOG) correction, which rejects the components of the neural recordings due to eye movements. Other techniques include band-pass and band-stop filters, to exclude common mode and high frequency bands that are likely to include more noise than real signal.

The filtered signals are then further processed to extract and select features for the BCI task. These include common spatial pattern (CSP) [21], principal component analysis (PCA), Riemannian geometry [22], average power of neural oscillations. Finally, these features are fed to a classification submodule, which includes a machine learning model that transforms such features into a BCI output.

### B. Control Interface

The Control Interface is responsible for the collection of sequences of LS from the transducer and for encoding them into Semantic Symbols (SS) used by the specific application. For example, in a P3Speller, two LSs, a row, and a column, are combined to select a character to be spelled (the SS). No brain signals are directly processed into the control interface.

The Logical Alphabet, then represents the interface between a Transducer and a Control Interface, so that different Transducers and Control Interfaces can be mixed and matched provided that their Logical Alphabets are compatible. It is clear that benchmarking a BCI system at the output of a Control Interface combines the classification accuracy and the ability of the Control Interface to prevent or correct errors.

**Fig. 4.**
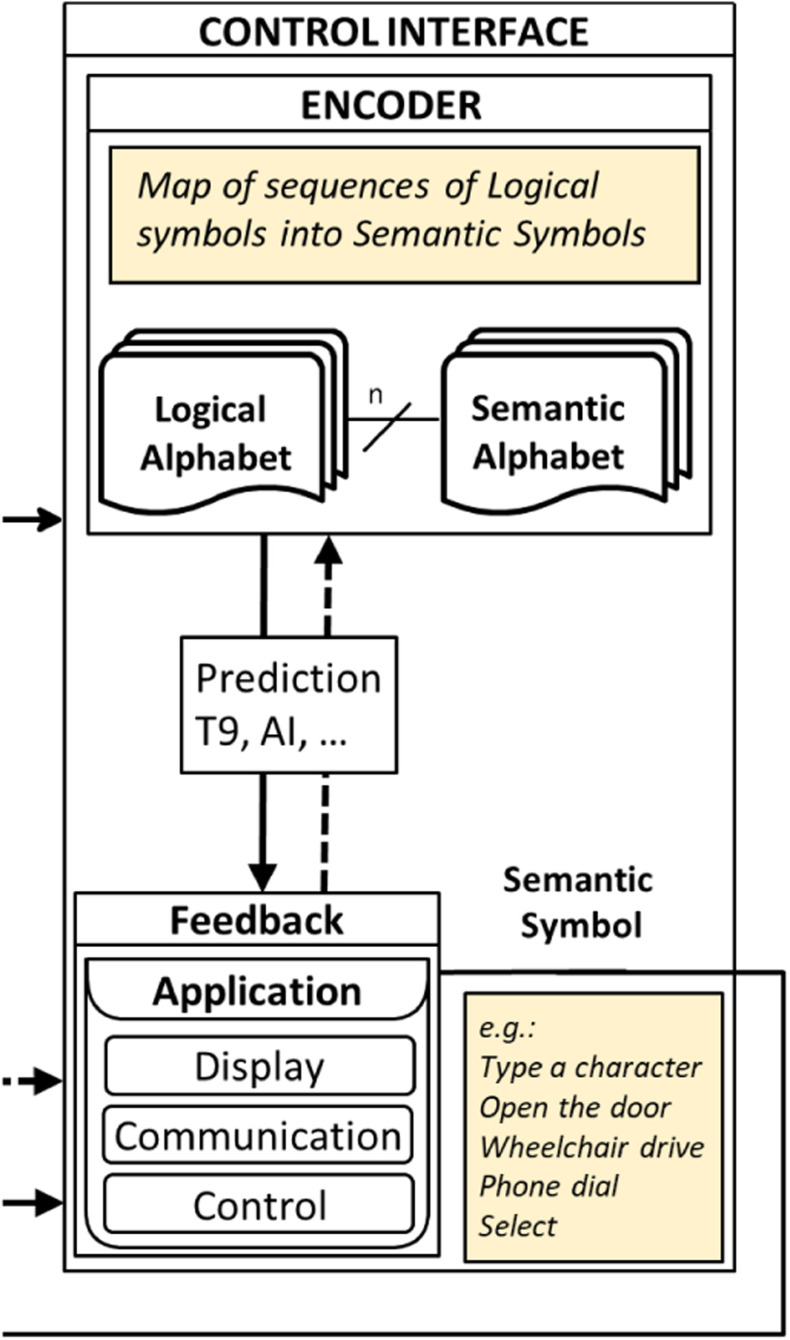
Control Interface.

The two contributions of the Transducer and the Control Interface should then be communicated separately. This will allow performance differentiation between the ability to detect brain patterns and the optimization of the encoding of logical into semantic alphabets. Some of the important control interface design considerations include the information transfer rate(bit rate), spatiotemporal resolution, calibration of electrode sensitivity, physiological delay, training needed, demand on attention, etc. The control interface portion of the P2731 working group's functional model is shown in Fig. 4.

Since the term “Brain–Computer Interface” was first coined by Vidal in 1973. Many interesting BCI systems have been developed for a wide variety of applications. BCIs can be categorized by the following dimensions [23]:

• Brain signal pattern (e.g., Steady State Somatosensory Evoked Potential (SS_EP), P300, ERD/ERS, Slow Cortical Potential (SCP))
• Stimulus modality (e.g., visual, auditory, tactile, self-regulated)
• Mode of operation (e.g., selective attention, cognitive efforts)
• Operation strategy (e.g., synchronous or cue-based, asynchronous or self-paced)
• Recording method (e.g., noninvasive, invasive)

As shown in Fig. 4, feedback is a part of the control interface. Typically, BCI also incorporates user feedback. This is a central part of the function of any BCI. The human user. Without feedback from the user, meaningful use of BCI, at least in many applications, is problematic if not impossible.

### C. Physiology

By their very nature, brain computer interfaces must interact not simply with neurophysiology, but with specific regions. and with specific individuals. A variety of factors influence whether BCI systems function properly with a particular individual, and many studies discard results from subjects who are unable to interface with the transducer. Classifying BCI-resistant subjects and developing, if possible, models for interfacing with them will be necessary for widespread deployment of BCI. Neurophysiology, as it relates to BCI, obviously focuses on brain waves . Having specific terminology for particular frequency ranges is critical to reporting and understanding research.

Considerable advances are being made in the classification of brain waves, and in understanding how they interact to produce physiologically meaningful signals, so it is necessary to maintain flexibility in future lexicons.

### D. Psychology

Physiological signals may vary also because of the subject's psychological state, such as motivation, depression, etc.. Furthermore, in both research and medical practice, BCI is often implemented in response to issues that have psychological connotations. BCI is frequently implemented in response to treatment for cognitive impairments as well as motor function impairments. Mental strategies such as motor imagery, relaxation or word association are often used to enhance transducer efficiency, and we expect explicit methods of subjective control to be further classified and used to enhance machine learning in future systems. Subject feedback is also affected by psychological issues. Feedback will affect classification as well as functionality.

## IV. Data Storage & Sharing

In recent years, it is common to find several data resources on the Web (data files, software tools, source code, etc.). However, it is also common that such data resources are not compatible with each other, in part because different file formats are used. The consequence of this is that it is necessary to write specific software for handling each dataset. This hinders the release software tools that could be used to analyze data from different datasets or that can automate certain procedures. The consequences of such a situation are that, despite the large availability of data sets, only a portion of these data sets can be amalgamated for assessment, thus reducing the statistical power of analyses.

BCI systems utilize a wide range of different control signals, recorded with diverse modalities (e.g., EEG, EMG, ECoG, LFPs, etc.) and that are stored in heterogeneous file formats, data organization and meta-data.

To improve BCI research, data should be shared in a manner that facilitates ease of use for other researchers to understand and use. In addition to a common format, specific data should be included. In particular, the following information should be reported: number of participants, details of the signal acquisition device, experimental conditions, signal processing pipeline and parameters. Data should be anonymized, and participants should have consented for their data to be shared.

Machine learning has been widely applied to biomedical research for several years. The use of machine learning also requires a common data format. There are currently training data sets for BCI available on the internet (e.g., Kaggle currently hosts 7 BCI datasets and 4 related challenges).

Given the high degree of heterogeneity of recorded control signals, it is difficult to identify a unique data format that is both efficient and complete. Often, the recorded signals are multidimensional (numerous electrodes, 2D/3D images, etc.) and with long durations. This necessitates an efficient and possibly lossless compression of the dataset. At the same time, the recorded signals have to be synchronized to the provided stimuli, for instance, for a P300 speller, the precise time instants of the stimuli presentation have to be included in the dataset shared.

The adoption of a standard file format would allow researchers to effectively share data [24]. Such a standardized format would also facilitate the release of software tools that can be widely used by the various stakeholders and to provide more reliable advancement in several research domains. A standardized data file format will improve research communication, collaboration, and analysis.

The FAIR principles (Findability, Accessibility, Interoperability, and Reuse of digital assets) were proposed to overcome this problem for pharmaceutical data [25]. As this issue also affects BCI research, it is appropriate to apply the FAIR principle to BCI data.

For the definition of a standard file format, it is necessary to meet the following basic criteria:

1) to have a common vision of a BCI, like the one described by a functional model;
2) to define what information should be stored, to allow data sharing without the need for additional documentation;
3) to choose the technology that should be used (e.g., XML, XDF, etc.). Such technology should allow extensibility to not break background compatibility if extensions in file formats should be required.

At present, the P2731 WG is addressing the first two issues. The second pre-requisite should be dealt with at different levels. In particular, the following levels are debated within the working group and should be considered preliminary and incremental, in the sense that each level extends the previous one:

a) Level 0 (brain signals): brain signals, sampling rate, sensors labels AND/OR location, and events. It also may be an appropriate place to store subject data. At this level, no BCI specific information is considered. These are the typical information provided by the manufacturer of the employed acquisition device. Other information, depending on the acquisition device should be also provided. For example, reference and ground sensors for EEG data, as well as power line frequency and sensor technology (e.g., dry vs. wet electrodes, active vs. passive, etc…) should be considered.
b) Level 1 (BCI training): This level provides details that fully describe the BCI paradigm should be included, such as the encoder (mapping of logical symbols into semantic ones), Inter Trial Interval, stimulation parameters, etc. No feedback driven by brain signals is required. In summary, all of the information needed to train a classifier should be included.
c) Level 2 (Feedback). If some feedback is provided during a trial (e.g., mu-rhythm), then the formula that drives it has to be provided because stored brain signals are assumed to be modulated by the feedback. Modulation by feedback can occur pre or post classification. At this level, two sub-cases should be distinguished to differentiate copy-mode vs. free-mode tasks. In the first case, true labels are available, while in the second case one can at least deduce them with a certain confidence. These cases can be referred to as Level 2C (for Copy mode) and Level 2F (Free run mode). Classified labels should be also provided.

In addition to the formatting of data storage, another key issue will be proper data acquisition. All stimulus, triggers, events, markers, and related data must be stored. Data classification is only truly meaningful in light of the concurrent data just enumerated. And of course, subject data must also be stored.

## V. Conclusion

The BCI community is especially diverse. This area of research is a nexus that is at the intersection of neuroscience, medicine, psychology, and various engineering disciplines. The diversity of backgrounds for researchers can be a hindrance to effective communication of research. Individual scientists and teams come to BCI from a wide range of educational and professional backgrounds. Each with its own terminology and processes. Inter-researcher communication and understanding is facilitated by a standardized glossary and a related functional model. The objective of the IEEE P2731 working group is to provide such a common lexicon and functional model.

In this paper we proposed a functional BCI framework to unify the different terminologies used in BCI research. Our aim is to help BCI researchers, clinicians, and practitioners design, develop and describe a BCI system, using shared terminology.

The proposed model has several limitations. First, it is a work in progress, and as such it is expected to be discussed and extended by the community. This presents the current status of the work of the IEEE P2731 working group. By publishing this work, the wider BCI community has an opportunity to review and perhaps provide input. We hope this functional model would help advance the BCI field in a more rigorous and time-efficient matter, reducing the burdens created by using different terminology.

There is still room for additional work. Incorporating hybrid BCI systems is one such area. Working on error correction being incorporated into the model is yet another area. These items, as well as continuing to refine the functional model as well as the glossary, will be addressed by the P2731 working group in the coming months.
